# A Carnitine-Containing Product Improves Aspects of Post-Exercise Recovery in Adult Horses

**DOI:** 10.3390/ani13040657

**Published:** 2023-02-14

**Authors:** Sally E. Johnson, Madison R. Barshick, Madison L. Gonzalez, Julia Wells Riley, Megan E. Pelletier, Beatriz C. Castanho, Elayna N. Ealy

**Affiliations:** School of Animal Sciences, Virginia Polytechnic Institute and State University, Blacksburg, VA 24061, USA

**Keywords:** equine, exercise, carnitine, recovery

## Abstract

**Simple Summary:**

Horses performing strenuous exercise can experience tissue damage, causing a delayed return to work. L-Carnitine supplements may accelerate the post-exercise recovery period, allowing for an earlier return to work. Adult Thoroughbreds were administered a commercial carnitine-containing recovery aid prior to performing exercise to exhaustion (D1). The activity was repeated after a single day of rest (D2), with physiological and biochemical measures obtained following exercise on both days. The results demonstrate that horses receiving the L-carnitine-containing supplement retained a greater range of motion in their fetlock on D2 than the controls. A normal inflammatory response to exercise was observed for all horses on D1, which was not found on D2; the heart rate recovery on D2 was also slower. These results indicate that a single day between strenuous work is insufficient to allow for tissue recovery.

**Abstract:**

Strenuous exercise can cause tissue damage, leading to an extended recovery period. To counteract delayed post-exercise recovery, a commercial product containing L-carnitine (AID) was tested in adult horses performing consecutive exercise tests to exhaustion. Fit Thoroughbreds were administered an oral bolus of placebo (CON) or AID prior to performing an exercise test to exhaustion (D1). The heart rate (HR) and fetlock kinematics were captured throughout the exercise test. Blood was collected before, 10 min and 1, 4 and 6 h relative to exercise for the quantification of cytokine (*IL*1*β*, *IL*8, *IL*10, *TNFa*) gene expression and lactate concentration. Horses performed a second exercise test 48 h later (D2), with all biochemical and physiological measures repeated. The results demonstrate that the horses receiving AID retained a greater (*p* < 0.05) amount of flexion in the front fetlock on D2 than the horses given CON. The horses presented a reduced (*p* < 0.05) rate of HR decline on D2 compared to that on D1. The expression of *IL*1*β*, *IL*8 and *IL*10 increased at 1 h post-exercise on D1 and returned to baseline by 6 h; the cytokine expression pattern was not duplicated on D2. These results provide evidence of disrupted cytokine expression, HR recovery and joint mobility in response to consecutive bouts of exhaustive exercise. Importantly, AID may accelerate recovery through an undetermined mechanism.

## 1. Introduction

Exertional exercise leading to fatigue is the culmination of multiple body systems experiencing a loss of functional capacity. Musculoskeletal fatigue presents as reduced contractile activity, buffering capacity and energy reserve depletion in the muscle and disrupted collagen fiber crimp patterns and stiffness within the tendon and ligament structures [1,2]. Numerous factors including age, sex fitness level and exercise intensity affect the timing of fatigue and recovery from the condition. Tendon microstructure disorganization and bone microfractures are a cumulative response to cycle numbers, duration and load, which can result in overload damage [3]. The early detection of physiological and material fatigue (bone, tendon, ligament) may prevent irreversible tissue damage and lengthy musculoskeletal repair.

Fit, trained horses display greater numbers and sizes of muscle mitochondria, which provide for an improved aerobic capacity, less of a reliance on glycogen and a shift toward fatigue resistance [4]. Race training increases complex I and 5 genes that are critical for mitochondrial activity and electron transport [5]. Improved oxidative capacity with training facilitates muscle glycogen sparing, with a concurrent lower amount of blood lactate observed [6]. Even with these exercise adaptations, the horse exhibits delayed post-exercise muscle recovery times, as indicated by the slower glycogen repletion and time to peak satellite cell activity in comparison to man [7,8,9]. Deep digital muscle fatigue at the gallop may lead to a greater load placed upon the superficial digit flexor tendon (SDFT), predisposing the structure to strain-induced damage and possible overload rupture [10]. Thus, dietary strategies that accelerate the process of muscle repair and recovery may provide the equine athlete with an opportunity to return to work sooner and offset the accumulation of tendon and joint damage.

Muscle is the largest reservoir of L-carnitine in the body. The lysine derivative participates as acylcarnitine in the transport of fatty acids into the mitochondria for β-oxidation while also serving to reduce free radical accumulation and oxidative stress damage [11]. Improved fatty acid flux through the mitochondria suggests that the molecule may be an ergonomic aid. However, chronic carnitine supplementation to fit men and women gives conflicting results, with both modest gains in athletic performance and no effects on speed or power [12,13]. Recent evidence supports carnitine ingestion as a post-exercise recovery aid, as recreationally active adults demonstrated lower blood creatine kinase levels and less muscle pain after a bout of exercise [14]. Although 10 weeks of supplementation to horses augments the training response of increased type IIA fibers [15], no studies report the ability of the molecule to modify the post-exercise recovery period.

Cytokine expression increases in the immediate post-exercise period. The pro-inflammatory response of human athletes includes elevated blood concentrations of interleukin (IL) 1β, IL6 and tumor necrosis factor α (TNFα), with the concentrations being dependent upon the exercise intensity [16]. The inflammatory response is necessary for the removal of muscle tissue damage and the initiation of the repair process [17]. Upon the completion of a 2 h bout of submaximal exercise, young, fit Quarter Horses demonstrate elevated systemic concentrations of IL1β, IL6, IL8, IL10 and TNFα within the first hour of recovery [18]. Similar results are observed during the post-race period in endurance horses with increased blood *IL*1*β*, *IL*8, *IL*10 and *TNFα* mRNA concentrations [19]. Reducing the pro-inflammatory response to exercise may expedite muscle repair and recovery in horses. Carnitine supplementation to mice with muscle wasting reduced the systemic concentrations of IL1 and IL6 and blunted the TNFα-stimulated myotube atrophy in vitro [20]. Similar anti-inflammatory effects occur in mice and rats suffering from cardiovascular disease [21], polycystic ovary disease [22] and artheroschlerosis [23]. Thus, the supplementation of carnitine to horses may decrease cytokine expression and accelerate post-exercise recovery.

The hypothesis that carnitine can dampen the post-exercise pro-inflammatory state, leading to an improved rate of recovery, was tested in fit adult horses.

## 2. Materials and Methods

### 2.1. Diet and Husbandry

Sixteen adult Thoroughbred horses (6.7 ± 0.5 yrs; 525 ± 10 kg) were maintained in groups of two or three on 0.2 ha dry lot paddocks and individually fed 0.25% of body weight concentrate (Ultium Competition, Land O’lakes, Inc., Arden Hills, MN, USA) and mixed grass hay at 1.75% of body weight (Table 1). The horses had unlimited access to water, salt and minerals. The nutrient content of the diet exceeded the requirements for adult horses in moderate work (NRC, 2007). Sixty minutes prior to performing an exercise test, each horse was administered a product containing 2.5 g acetyl L-Carnitine HCl, 1.5 g L-carnitine tartrate, 2.5 g sodium chloride, 2.0 g L-glycine, 1.5 g L-leucine, 1 g ascorbic acid, 500 IU d-alpha tocopheryl acetate and 1 g alpha lipoic acid (Platinum Renew, Platinum Performance, Buellton, CA, USA) in 30 mL of unsweetened applesauce. The same product was administered 60 min after the completion of the exercise test. The control horses received applesauce only. A second exercise test was performed 48 h later without the administration of the recovery aid product.

### 2.2. Training and Exercise Test

The horses performed exercise on a motorized hot walker three times per week for 6 weeks. The daily training workload was two sets of 2 m/s for 5 min, 4 m/s for 11 min and 9 m/s for 5 min, with a change in direction between sets. After 6 weeks of training, the horses performed an incremental exercise test to exhaustion on a high-speed treadmill (EquiGym, Lexington, KY, USA). The test parameters were 4 m/s for 4 min and 10 m/s for 1 min, with sequential 1 m/s/min increases until the horse was unable to maintain its position on the treadmill, at which time the speed was reduced to 4 m/s for 5 min. All exercises were performed on a three-degree incline. The horses repeated the exercise test (D2) 48 h after the completion of the first test (D1).

### 2.3. Heart Rate Analysis

Heart rate (HR) was recorded throughout the exercise test with a wearable sensor (V800, Polar, Bethpage, NY, USA). The time and speed to reach 200 bpm (V200), maximum HR (HRmax) and total gallop time (10 m/s to fatigue) were extracted from the recorded data. The heart rate from fatigue to the end of the cool-down period (5 min) was plotted against time and analyzed by nonlinear regression for the calculation of the HR recovery for each horse.

### 2.4. Sample Retrieval

Venous blood from the jugular was collected into a lithium heparin or an EDTA-containing tube (8 mL/tube) before exercise and 5 min and 1, 4 and 6 h post-exercise. Plasma (heparin tubes) was harvested following centrifugation at 1500× *g* for 10 min at 4 °C and stored in aliquots at −80 °C until the analysis (<30-days). Plasma lactate was measured colorimetrically using a standard curve of known concentrations, according to the manufacturer’s recommendations (MAK064, Sigma Aldrich, St. Louis, MO, USA). Whole blood (2 mL) was transferred immediately from the EDTA-containing blood tube and mixed with 6 mL RNALater prior to storage at −20 °C for RNA isolation. Total RNA was isolated by lysis and centrifugation through glass filter spin columns (RiboPure RNA, blood, ThermoFisher, Waltham, MA, USA) and stored frozen at −80 °C until use.

### 2.5. Quantitative Reverse-Transcription Polymerase Chain Reaction (qRT-PCR)

Genomic DNA contaminates were removed from the RNA isolates (Ambion DNase, ThermoFisher, Waltham, MA, USA) prior to reverse transcription (High-Capacity cDNA RT kit, Applied Biosystems, ThermoFisher, Waltham, MA, USA). Five nanograms of cDNA were amplified with gene-specific primers (Table 2), thermal stable DNA polymerase and SYBR chemistry (Power SYBR Master mix, Applied Biosystems, ThermoFisher, Waltham, MA, USA) in an Aria Mx thermocycler (Agilent, Santa Clara, CA, USA) for 40 cycles of 95 °C for 15 s and 60 °C for 60 s. All primer efficiencies were greater than 90%. Melt curve analysis revealed a single amplicon for all primer sets. Glyceraldehyde 3-phosphate dehydrogenase was used as the internal normalization control. The relative expression was calculated as 2^−ΔΔCt^ with ΔΔCt = ΔCt (gene of interest) − ΔCt (pre-exercise D1, control group average).

### 2.6. Kinematics

Video of the gallop portion (≥10 m/s) of the exercise test was recorded with a Quintic High-Speed LIVE camera (Quintic Consultancy Ltd., Birmingham, UK) using an f/1.2, 8 to 48 mm zoom lens (Computar H6Z0812, CBC America, Cary, NC, USA) at a capture rate of 150 frames per second. The posterior angle of the right front fetlock at the stance phase of the gait cycle was measured for five strides at 10 m/s and at fatigue using Quintic Biomechanics Video Analysis Software (v.31, Quintic Consultancy Ltd., Birmingham, UK).

### 2.7. Statistics

The data were analyzed as a three-way ANOVA, with the day of exercise, treatment, time relative to exercise and interactions as the fixed variables. In the absence of a treatment effect, the data were combined and analyzed by two-way ANOVA, with the day, time and day X time as the main effects. Sidak’s multiple comparison test with a pooled variance was performed, with the significance established at *p* < 0.05.

## 3. Results

Treatment with AID prior to and after an incremental exercise test did not affect the time to reach V200, time to HRmax, time spent at HRmax, total gallop time or HR recovery rate (Table 3). The decline in HR during the recovery period was slower (*p* = 0.01) on D2 than it was on D1 (Table 3).

**Figure 1:**
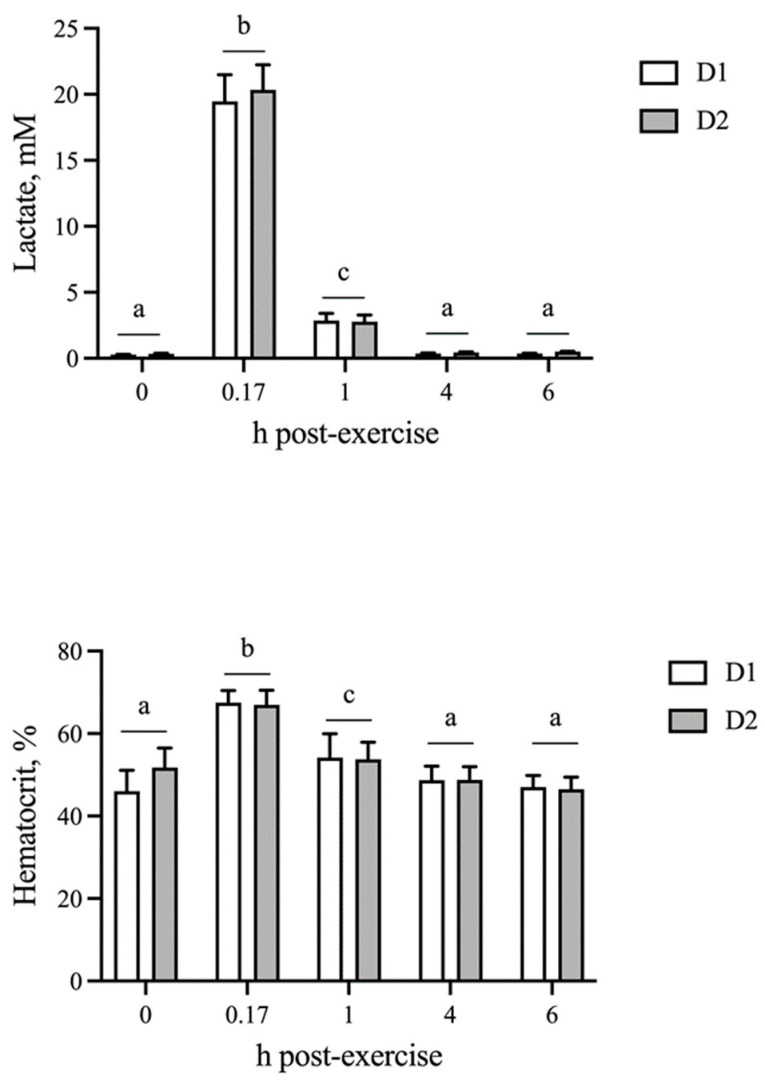
Blood lactate and hematocrit responses following sequential bouts of exercise. Blood lactate and hematocrit increase at 10 min post-exercise, followed by a decline to baseline by 4 h on both days of exercise. Means and SEMs are shown. Different letters indicate significance at *p* < 0.05.

The blood lactate concentration and hematocrit were unchanged by AID (*p* = 0.37 and *p* = 0.70, respectively). As expected, both physiological parameters were elevated (*p* < 0.05) at 10 min post-exercise and returned to baseline by 4 h on both D1 and D2, which did not differ from one another (Figure 1).

**Figure 2:**
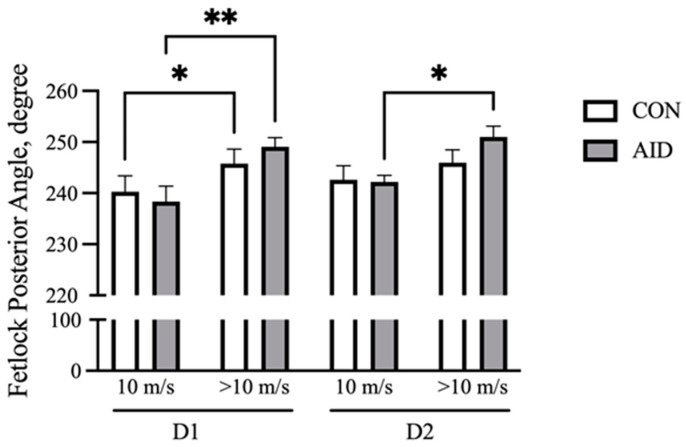
AID facilitates the retention of the fetlock range of motion. Horses performed consecutive bouts of exercise, with posterior fetlock angle measures recorded via high-speed cameras. The posterior angle of the right front increased over the course of the gallop on day 1 (D1) of exercise for both groups. AID horses retained a greater range of motion on day 2 (D2) of exercise. * denotes significance at *p* < 0.05, and ** denotes significance at *p* < 0.01.

With increasing speed, the front fetlock posterior angle at the full stance phase increases and represents the flexion at the metacarpal phalangeal (MCP) joint [27]. Fetlock angles were analyzed at the beginning of the gallop (10 m/s) and at fatigue (>10 m/s). The results demonstrate that both CON and AID experienced an increase (*p* < 0.05) in the flexion over the course of the gallop on D1 (Figure 2). The horses receiving the AID experienced an increase (*p* < 0.05) in the posterior angle during the gallop on D2, while CON did not demonstrate a significant increase in the flexion.

The inflammatory gene expression was not influenced by AID; thus, the data were consolidated. For all of the genes examined, the post-exercise expression levels (*p* ≤ 0.01) were affected by time (Table 4). The relative expression of *IL-*1*β* and *IL-*8 increased (*p* < 0.05) within 1 h post-exercise, with a return to baseline levels by 4 and 6 h, respectively (Table 4). The expression of *IL-*1*β* and *IL-*8 was greater (*p* < 0.05) on D1 than it was on D2, largely owing to the little change in their expression during the post-exercise period of D2 (Table 4). Divergent *IL-*10 expression patterns were noted following the two exercise tests. On D1, a 60% increase in the relative *IL*10 mRNA abundance at 1 h post-exercise was observed, which declined (*p* < 0.05) to baseline by 4 h (Table 4). By contrast, equivalent amounts of *IL*10 mRNA were measured at the pre-exercise and 1 h post-exercise timepoints on D2, which declined (*p* < 0.05) to levels less than those found at either the pre-exercise or 1 h post-exercise timepoints. A small decrease (*p* < 0.05) in *TNFɑ* mRNA abundance was observed at 4 h post-exercise in comparison to that at the pre-exercise timepoint (Table 4).

## 4. Discussion

L-carnitine participates in the transportation of fatty acids into the mitochondria, where they undergo β-oxidation for energy production. The long-term supplementation (<7 days) of carnitine is proposed to increase the muscle carnitine content, which leads to improved fatty acid oxidation efficiency and, possibly, athletic performance [28]. By contrast, the ingestion of L-carnitine by young men one hour prior to exhaustive treadmill exercise reduced lipid peroxidation measures and increased blood concentrations of glutathione, suggesting that the dipeptide works as an anti-oxidant [29]. Although training alone improves the redox status in horses [30,31,32], adult polo horses supplemented with the antioxidant vitamin E demonstrated an increase in muscle MyoD mRNA abundance 4 h after exercise, suggesting accelerated recovery and repair [33]. A primary objective of the experiment was to determine if a carnitine-containing product could offset fatigue-induced performance detriments, facilitating a faster return to work. Sequential exercise tests with an intervening window of a single day of rest were selected as a stressor because others have reported that muscle glycogen repletion can take up to 72 h [7]. With reduced glycogen stores, the expectation was that horses would reach fatigue sooner on D2, with less time spent on the gallop. The lack of a difference in any performance parameter between the two exercise tests indicates that deficits in muscle metabolism were unlikely; thus, an AID effect would be difficult to measure. This does not eliminate insufficiencies in other physiological systems, however, as fatigue is caused by multisystem functional reductions. The inability of post-exercise HR to recover at equivalent rates on day 1 and day 2 support continued biological limitations due to strenuous exercise. The AID did have an effect on MCP joint mobility, indicating that the carnitine-based product does offer some post-exercise relief. Tendon damage is a product of the cycle number and load. The accumulation of microdamage leading to the fatigue failure of the superficial digit flexor tendon occurs at lesser strain loads in older horses in comparison to young adults [34]. A greater retention of the MCP flexion may partially alleviate tendon and ligament strain and joint stiffness, thus offering some protection against repetitive cyclic stress. These interpretations are speculative, however, as neither the muscle metabolism nor the pre-AID supplementation of the treatment horses were measured.

Both horses and men experience muscle damage following bouts of intense exercise, which is characterized by an increase in blood creatine kinase [35,36,37]. During the initial 4 h post-exercise, neutrophils and pro-inflammatory macrophages infiltrate the muscle tissue to begin the repair process [38]. These cells produce and secrete cytokines (TNFɑ, IL6, IL10, 1L1β) that work in concert with myokines to stimulate satellite cell activation, myotube formation and metabolic recovery. Following a bout of exhaustive exercise, adult horses demonstrate an increase in the blood concentrations of *IL*1*β*, *IL*6 and *TNFα* mRNA [24,39]. The levels remain elevated at 24 h post-exercise in comparison to the pre-exercise values. Young, fit horses performing a single bout of submaximal exercise demonstrated greater serum concentrations of IL1*β*, IL6, IL8, IL10 and TNFɑ, with transient declines toward baseline concentrations within 6 h for many of the cytokines [18]. Although the level of exercise required to initiate the inflammatory responses is unknown, the timeline and global cytokine response are comparable between submaximal and strenuous exercise. Our results following the first bout of exercise are consistent with these and demonstrate increased amounts of *IL*1*β*, *IL*8 and *IL*10 mRNA in comparison to pre-exercise levels. However, an increase in *IL*1*β* and *IL*8 was not observed following the second bout of exercise. Both cytokines are produced primarily by monocytes and macrophages [40]. Following the first bout of exercise, the macrophages likely infiltrated the muscle tissue to begin the damage repair process. This would deplete the circulating monocyte and macrophage population, leading to diminished *IL*1*β* and *IL*8 mRNA detection in the blood. These results indicate that sequential bouts of exercise performed within 48 h of one another are detrimental and provide insufficient recovery time to mount a full inflammatory response. In turn, this is likely to prolong the recovery period and may leave the equine athlete susceptible to infection and other immune insults.

Exercise causes an increase in blood mononuclear cell TNFα mRNA expression and an increase in the amounts of the cytokine protein in the peripheral blood of horses [41,42]. By contrast, others report no differences between pre- and post-exercise *TNFα* mRNA content in the blood [43]. Our results demonstrate a minor reduction in the *TNFα* mRNA content with time, which offers limited biological significance. The disparity in the results can likely be attributed to the time of sample acquisition and the differences in animal numbers. Because there was no treatment effect, the horse numbers were larger than those reported in other studies [42,44]. Equally importantly, the training status of the horses may have impacted the cytokine expression levels. Healthy young men participating in a resistance exercise program demonstrated no changes in the serum TNFα concentration within the first 6 h of recovery [45]. Peripheral lymphocytes isolated from fit men following exercise failed to increase TNFα production in response to an inflammatory stimulus in vitro [46]. After 6 weeks in a moderate exercise training regimen, the horses were considered fit and may not invoke an increase in TNFα following intense work. The importance of TNFα to the recovery period may also be restricted to the affected tissue, as an increase in the expression of the cytokine in the skeletal muscle was apparent immediately following exercise in comparison to the peak expression of the cytokine at 6 h in the peripheral blood cells of unfit adult horses [44]. Future efforts should examine the levels of the pro-inflammatory mediator in the skeletal muscle of fit adult horses to add context to the expression pattern and the role of the cytokine during exercise-induced damage repair.

## 5. Conclusions

In conclusion, a carnitine-containing recovery aid may elicit a positive response with respect to post-exercise recovery in horses. The aid, however, does not offer any modulatory benefits for the systemic blood pro-inflammatory response to exercise. The results of this study point to the importance of adequate rest periods between exercise bouts in ensuring that sufficient macrophages and monocytes are available for tissue repair.

## Figures and Tables

**Table 1:** Chemical composition of feedstuffs; dry matter basis.

Component	Grain	Hay
DE, Mcal/kg	3.33	2.11
Crude protein, %	13.9	16.5
Crude fat, %	14.9	4.0
Lysine, %	0.7	0.6
Acid detergent fiber, %	22.4	35.6
Neutral detergent fiber, %	34.4	60.8
Calcium, %	1.2	0.46
Phosphorus, %	0.56	0.30
Magnesium, %	0.39	0.31
Potassium, %	1.30	2.07
Sodium, %	0.20	0.13
Iron, ppm	1130.0	112.0
Copper, ppm	44.0	9.0
Manganese, ppm	211.0	64.0
Zinc, ppm	195.0	26.0
Molybdenum	1.5	1.2

**Table 2:** Primer sequences.

Gene ^1^	Accession Number	Forward, 5′-3′	Reverse, 5′-3′	Reference
*IL*1*β*	NM_001082526.1	GTCTTGGAAGCTGCCCTTCA	GTCTTGGAAGCTGCCCTTCA	[24]
*IL*8	NM_001083951.2	CTGGCTGTGGCTCTCTTG	CAGTTTGGGATTGAAAGGTTTG	[25]
IL10	NM_001082490.1	TGTTGTTGAACGGGTCCCTG	ACTCTTCACCTGCTCCACTG	[25]
*TNFɑ*	NM_001081819.2	AAGGACATCATGAGCACTGAAAG	CGGCCCCCTGCCTTCT	[24]
*GAPDH*	NM_001163856.1	CCACCCCTAACGTGTCAGTC	AATCGCAGGAGACAACCTGG	[26]

^1^ IL1β, interleukin 1β; IL8, interleukin 8; IL10, interleukin 10; TNFɑ, tumor necrosis factor ɑ; GAPDH, glyceraldehyde 3-phosphate dehydrogenase.

**Table 3:** Heart rate (HR) and performance parameters for adult horses consuming a placebo (CON) or recovery aid (AID) and completing a standardized exercise test to fatigue at 48 h intervals ^1^.

		CON			AID			*p*-Value		
		Mean		SEM	Mean		SEM	D	A	D X A
HRmax, bpm	D1	214.0	±	3.2	221.6	±	3.5	0.67	0.09	0.90
	D2	214.5	±	2.7	222.3	±	3.4			
Time to HRmax, s	D1	232.9	±	19.8	233.4	±	24.9	0.16	0.86	0.94
	D2	206.6	±	15.5	212.3	±	23.7			
Time at HRmax, s	D1	13.3	±	5.4	58.6	±	32.7	0.62	0.31	0.13
	D2	19.9	±	5.8	77.1	±	34.0			
Time to V200, s	D1	74.9	±	19.5	79.9	±	26.8	0.44	0.92	0.39
	D2	71.1	±	14.4	59.0	±	18.2			
Gallop time, s	D1	243.1	±	15.1	275.6	±	11.6	0.12	0.14	0.94
	D2	236.3	±	17.3	268.1	±	17.4			
Recovery HR decline, bpm/m	D1	−43.8	±	4.0	−42.9	±	4.3	0.01	0.70	0.24
	D2	−35.4	±	3.8	−39.9	±	3.7			

^1^ Exercise test was performed at day 1 (D1) and repeated after 48 h (D2). D, day; A, AID; D X A, day by AID interaction.

**Table 4:** Relative expression of cytokine mRNA before (0) and 1, 4 and 6 h post-exercise.

Gene	Time ^1^, h	Day 1			Day 2			*p*-Value		
								Day	Time	D X T ^2^
*IL*1*β*	0	1.08	±	0.10 ^a^	0.89	±	0.22 ^x^	0.03	≤0.01	0.12
	1	1.81	±	0.28 ^b^	1.09	±	0.18 ^y^			
	4	1.90	±	0.21 ^b^	1.10	±	0.26 ^y^			
	6	1.34	±	0.12 ^a,b^	0.89	±	0.19 ^x,y^			
*IL*8	0	1.11	±	0.12 ^a^	1.16	±	0.25 ^x^	0.03	0.02	0.06
	1	2.29	±	0.46 ^b^	1.35	±	0.25 ^y^			
	4	2.10	±	0.25 ^b^	1.09	±	0.18 ^x,y^			
	6	2.04	±	0.29 ^b^	1.15	±	0.22 ^y^			
*IL*10	0	1.72	±	0.27 ^a^	1.65	±	0.36 ^ax^	0.24	≤0.01	0.02
	1	2.86	±	0.46 ^b^	1.68	±	0.41 ^ay^			
	4	1.17	±	0.15 ^ac^	0.81	±	0.19 ^bz^			
	6	1.12	±	0.20 ^ac^	0.99	±	0.21 ^bz^			
*TNFα*	0	1.05	±	0.09	1.02	±	0.28 ^x^	0.84	≤0.01	0.84
	1	0.95	±	0.09	0.82	±	0.21 ^x,y^			
	4	0.76	±	0.10	0.94	±	0.24 ^y^			
	6	0.95	±	0.09	1.13	±	0.24 ^x^			

^1^ Time post-exercise, with 0 representing pre-exercise. ^2^ D X T, day X time interaction. ^a,b,c^ within-column significant difference. ^x–z^ row mean (time) significant difference.

## Data Availability

The data presented in this study are available on request from the corresponding author.
